# Development of a reliable clinical assessment tool for meningoencephalitis in dogs: The neurodisability scale

**DOI:** 10.1111/jvim.16717

**Published:** 2023-04-24

**Authors:** Rita Gonçalves, Thomas W. Maddox, Stephanie Phillipps, Aran Nagendran, Camilla Cooper, Rocio Orlandi, Rory Fentem, Gemma L. Walmsley

**Affiliations:** ^1^ Department of Veterinary Science, Small Animal Teaching Hospital University of Liverpool, Leahurst Neston Cheshire United Kingdom; ^2^ Department of Musculoskeletal and Ageing Science, Institute of Life Course and Medical Sciences University of Liverpool Liverpool United Kingdom

**Keywords:** dog, MUO, NDS, outcome, prognosis

## Abstract

**Background:**

Meningoencephalitis of unknown origin (MUO) comprises a group of debilitating inflammatory diseases affecting the central nervous system of dogs. Currently, no validated clinical scale is available for the objective assessment of MUO severity.

**Objectives:**

Design a neurodisability scale (NDS) to grade clinical severity and determine its reliability and whether or not the score at presentation correlates with outcome.

**Animals:**

One hundred dogs with MUO were included for retrospective review and 31 dogs were subsequently enrolled for prospective evaluation.

**Methods:**

Medical records were retrospectively reviewed for 100 dogs diagnosed with MUO to identify the most frequent neurological examination findings. The NDS was designed based on these results and evaluated for prospective and retrospective use in a new population of MUO patients (n = 31) by different groups of independent blinded assessors, including calculation of interobserver agreement and association with outcome.

**Results:**

The most common clinical signs in MUO patients were used to inform categories for scoring in the NDS: seizure activity, ambulatory status, posture and cerebral, cerebellar, brainstem, and visual functions. The intraclass correlation coefficient (ICC) for prospective use of the NDS was 0.83 (95% confidence interval [CI], 0.68‐0.91) indicating good agreement, and moderate agreement was found between prospective and retrospective assessors (ICC, 0.71; 95% CI, 0.56‐0.83). No association was found between NDS score and long‐term outcome.

**Conclusions and Clinical Importance:**

The NDS is a novel clinical measure for objective assessment of neurological dysfunction and showed good reliability when used prospectively in MUO patients but, in this small population, no association with outcome could be identified.

**Abbreviations**
CNS: central nervous system
COM: clinical outcome measure
ICP: intracranial pressure
ICU: intensive care unit
MRI: magnetic resonance imaging
MUO: meningoencephalitis of unknown origin
NDS: neurodisability scale
NE: necrotizing encephalitis
SE: status epilepticus

## 1 INTRODUCTION

Meningoencephalitis of unknown origin (MUO) in dogs comprises a diverse group of idiopathic inflammatory diseases that can only be differentiated and confirmed on histopathology. This term includes the subtypes granulomatous meningoencephalomyelitis, necrotizing meningoencephalomyelitis, and necrotizing leukoencephalitis.^1^, ^2^, ^3^ The etiology of MUO is unknown, but it is considered most likely a group of immune‐mediated diseases considering their generally positive response to immunosuppressive treatment.^2^ The incidence of MUO in the canine population is unknown, but it has been reported as the most common inflammatory disease affecting the central nervous system (CNS) in dogs in a referral hospital population.^4^

Meningoencephalitis of unknown origin in dogs is a debilitating disease and, despite appropriate treatment, 25% to 33% of dogs die within a week of diagnosis.^5^, ^6^ Several studies have evaluated short and long‐term outcome in dogs with MUO, but all have focused on survival at different time points.^5^, ^6^, ^7^, ^8^, ^9^, ^10^, ^11^, ^12^, ^13^, ^14^ The use of survival as an outcome measure for MUO patients is flawed because affected dogs often are euthanized, and owners may elect for euthanasia at different time points based on a variety of complex underlying factors including financial constraints, difficulties managing chronic disease and views on what constitutes acceptable quality of life. We are therefore in need of an objective scoring tool specific for MUO that describes the general status of each patient, is easy to use and is repeatable. Such an instrument could improve our ability to monitor this condition, especially when studying the effects of different treatment protocols, and may assist in early prognostication. One previous study attempted to create an outcome score based on neurological deficits, but it was generated retrospectively and no attempts were made to validate or assess reliability of this score.^15^ The importance of developing objective scales that can guide physicians' daily practice as well as facilitate development of newer therapeutic options has been recognized in human patients with inflammatory diseases affecting the CNS.^16^, ^17^ Diseases such as multiple sclerosis (MS) and autoimmune encephalitis (AE) share some similarities with MUO.^18^, ^19^ In recent decades, several outcome measures have been developed that describe the clinical severity and functional deficits in patients with MS and AE. The most widely used measure is the Expanded Disability Status Scale (EDSS) for MS and the Clinical Assessment Scale in Autoimmune Encephalitis (CASE) for AE.^17^, ^20^, ^21^

Our aims were to: (1) design a disability scale that could be used to describe disease status; (2) evaluate the interobserver agreement of the NDS in dogs diagnosed with MUO prospectively and retrospectively; and (3) determine if the NDS score at initial presentation was associated with clinical outcome.

## 2 MATERIALS AND METHODS

### 2.1 Neurodisability scale design

To aid in the design of the disability scale, the medical records of 100 dogs consecutively diagnosed with MUO at the Small Animal Teaching Hospital (SATH) of the University of Liverpool were retrospectively reviewed. Ethical approval for use of data was granted by the Ethics Committee of the University of Liverpool (VREC805). Dogs included in the study met the following criteria: >6 months of age; multiple, single, or diffuse intra‐axial hyperintensities on T2‐weighted magnetic resonance (MR) images; mononuclear pleocytosis on cerebrospinal fluid (CSF) analysis (when performed); and negative serology for *Toxoplasma gondii* and *Neospora caninum*.^22^ Dogs were excluded if no pleocytosis was found on CSF analysis, except for dogs with signs of increased intracranial pressure (ICP) on imaging studies because CSF was not collected in these cases. The findings of the neurological examination performed on initial presentation were retrieved from the medical records and spectrum and frequencies of the different clinical signs in patients with MUO were determined.

The NDS was designed by a veterinary neurology diplomate (RG) and included only signs of neurological dysfunction that had been identified in the initial cohort of MUO patients. The EDSS and CASE scales used in humans were consulted because they are widely used instruments to assess disease progression in MS and AE and have been validated as useful primary outcome indicators in clinical trials. All members of the neurology team (including 4 veterinary neurology diplomates and 3 veterinary neurology residents in training) then were consulted, and minor changes made after review and recommendations.

The NDS (Table S1) was designed by attributing a numerical rating of dysfunction (0‐3, with the higher number denoting more dysfunction) for the following categories: seizures, ambulatory status, cerebral functions, cerebellar functions, brainstem functions, visual functions, and postural abnormalities. We assigned specific clinical signs identified most commonly in the initial population to the different degrees of dysfunction in each category so as to minimize subjectivity in the grading. The degree of disability and perceived effect on quality of life were taken into account when deciding on the degree of dysfunction to attribute to each deficit, similar to the use of the human scales, which usually describe whether or not a deficit affects ability to perform daily activities. Two binary categories also were added to include abnormalities identified in the initial cohort: presence or absence of hyperesthesia and presence or absence of proprioceptive deficits. The NDS score was calculated as the sum of the individual scores for each category.

### 2.2 Reliability of the NDS

A second population of dogs was prospectively enrolled and consisted of dogs presented to the SATH after development of the NDS and diagnosed with MUO following the same inclusion criteria. Each dog was examined on initial presentation by 2 independent blinded observers, which included board‐certified veterinary neurologists, veterinary neurology residents, or rotating interns. Each dog underwent a complete neurological examination including subjective gait analysis, postural reaction testing, cranial nerve examination, spinal reflex assessment, and paraspinal palpation. Each observer then independently completed the NDS standard sheet explaining the scale and assigned a total overall score. Access to clinical history, namely if seizures had occurred in the previous 7 days, was provided to both observers.

Furthermore, to assess the reliability of the scale through use of retrospective records for clinical research, 2 additional independent observers used the blinded medical records of the same prospectively enrolled dogs to retrospectively assign an NDS score at a separate time.

Outcome was assessed at the last follow‐up appointment or telephone conversation with the owners, but dogs that were euthanized without adequate treatment or lost to follow‐up in the initial 6 months after diagnosis were excluded from outcome analysis. Outcome was defined as good if clinical signs completely resolved and no relapse had been reported, fair if dogs had signs of relapse but responded to treatment changes (marked improvement after increased immunosuppression with minimal or no neurological deficits), and poor if dogs died from the disease or had signs of relapse with minimal to no response to changes in the treatment protocol (resulting in euthanasia).

Statistical analysis was performed using the software SPSS 27.0 (SPSS Inc, Chicago, Illinois). Continuous data were assessed for normality using the Shapiro‐Wilk test. Descriptive statistics are reported for continuous variables using mean (SD) for approximately normally distributed variables, median (interquartile range [IQR]) for variables with skewed distributions, and frequencies (with 95% confidence intervals [CI] where appropriate) for categorical variables.

Interobserver reliability was calculated using Cohen's kappa for binary categories (classified as absent or present), weighted kappa for ordinal categories (ranked 0‐3) of the scale, and the intraclass correlation coefficient (ICC) for the total scores. Intraclass correlation coefficient estimates and their 95% CI were calculated based on absolute agreement, 2‐way random effects models as a measure of prospective and retrospective interobserver agreement. All scores (2 prospective and 2 retrospective) then were used to calculate the ICC as measurement of interobserver agreement between prospective and retrospective use of the NDS.

A Kruskal‐Wallis test was used to evaluate the association between the NDS score and outcome. The Mann‐Whitney test was used to assess the associations between the NDS score and survival to discharge and the NDS score and relapse. The same test was used to evaluate the association between time to relapse and response to treatment at time of relapse. The correlation between NDS score and time spent in the intensive care unit (ICU) was assessed using the Pearson correlation coefficient.

## 3 RESULTS

### 3.1 Development of the NDS

The initial population used to develop the NDS consisted of 56 females (40 spayed) and 44 males (25 neutered). Median age at presentation was 48 months (IQR, 23‐83). Affected breeds were crossbreeds (n = 17), Pug (n = 11), French bulldog, West Highland white terrier and Shih Tzu (n = 5), Chihuahua, Yorkshire terrier and Staffordshire bull terrier (n = 4), Pomeranian, Jack Russell terrier and Labrador (n = 3), Lhasa Apso, miniature Poodle, miniature Schnauzer and English springer spaniel (n = 2), and 1 each of the following breeds: Chinese crested, Manchester terrier, Patterdale terrier, Irish terrier, Welsh springer spaniel, Lurcher, Golden Retriever, Flat Coat retriever, and English setter. The signs of neurological dysfunction reported in the clinical records are presented in Table 1 and were used to design the NDS (Table S1). In 6 of these dogs, CSF analysis was not performed because herniation through the foramen magnum was identified on MR images.

**TABLE 1:** Clinical signs identified in 100 dogs diagnosed with meningoencephalitis of unknown origin.

Clinical sign	% dogs affected
Proprioceptive deficits	70
Obtundation	60
Ataxia	55
Visual deficits	53
Head tilt	36
Seizures^a^	35
Paresis	29
Compulsive circling	28
Hyperesthesia	23
Behavior changes	18
Pathological nystagmus	17
Hypermetria	15
Head turn	14
Cranial nerve dysfunction	12
Tremors	10
Others^b^	29

^a^ Of the 35 dogs presenting with seizures, 26 had generalized and 9 focal seizures. Cluster seizures were reported in 21 dogs and status epilepticus in 4 dogs in the 7 days before presentation.

^b^ This category included positional strabismus (n = 16), reduced physiological nystagmus (n = 8), anisocoria (n = 3), incontinence (n = 1), and Horner syndrome (n = 1).

### 3.2 Reliability of the NDS

Thirty‐one consecutive prospectively enrolled patients diagnosed with MUO were assessed on initial presentation by 2 independent assessors. These included 4 European College Veterinary College (ECVN) diplomates (27 assessments), 4 ECVN residents (29 assessments), and 5 rotating interns (6 assessments). This population of dogs included 15 females (8 spayed) and 16 males (8 neutered). Affected breeds included crossbreeds (n = 7), Chihuahua (n = 6), French bulldog, pug and Yorkshire terrier (n = 3), Boston terrier, Jack Russell terrier (n = 2), and 1 each of the following: Lhasa Apso, Shih Tzu, Labrador retriever, Flat Coat retriever, and Newfoundland. Median age at presentation was 41 months (IQR, 24‐69). Over the 48 hours before presentation, 10 dogs had received nonsteroidal anti‐inflammatory drugs, 4 had received corticosteroids, 4 antimicrobials, and 4 antiepileptic medications. In 5 dogs, CSF analysis was not performed because herniation through the foramen magnum was identified on MR images.

The mean total NDS score at presentation was 8 ± 2.85. No patients achieved the theoretical minimum point value or the theoretical maximum point value. All dogs received immunosuppressive doses of dexamethasone and an 8‐hour constant rate infusion (CRI) of cytosine arabinoside immediately (200 mg/m^2^) after diagnosis. All 27 dogs that survived to discharge received long‐term treatment with prednisolone (started at immunosuppressive doses and then slowly tapered) which was used alone in 3 dogs or along with other immunosuppressive medications in the remainder. Additional immunomodulatory drugs were cytosine arabinoside administration every 3‐4 weeks (13), cyclosporine (7), or leflunomide (5). In the dogs that received cytosine arabinoside, it was eventually discontinued in 12 dogs (median, 6 administrations; IQR, 5‐8) and replaced by leflunomide in 3 dogs, cyclosporine in 2 dogs, and mycophenolate mofetil in 1 dog. A third medication was added to the treatment protocol in 4 dogs after clinical signs of relapse: cyclosporine (2) or procarbazine (2).

The reproducibility analysis results for the individual categories are summarized in Table S2. One category showed almost perfect interobserver agreement (seizures), 5 showed substantial interobserver reliability (ambulatory status, postural abnormalities, cerebral functions, cerebellar functions, and visual functions) and 1 showed moderate interobserver reliability (brainstem functions). The 2 binary categories showed no to slight interobserver reliability (presence or not of proprioceptive deficits) or only fair interobserver reliability (presence or not of hyperesthesia). For this reason, the latter 2 were excluded from the final NDS presented in Table 2.

**TABLE 2:** Final canine meningoencephalitis neurodisability scale.

	Score
Ambulatory status	
Normal	0
Mild paresis or ataxia present but ambulatory without falling	1
Moderate/severe paresis or ataxia present with frequent falling	2
Nonambulatory	3
Cerebral functions	
Normal	0
Disorientation, obtundation, or behavior changes (only 1 of these abnormalities)	1
2 or all the following: disorientation, obtundation, or behavior changes	2
Stupor, coma, compulsive circling, and/or head pressing (±disorientation, behavior changes, or obtundation)	3
Cerebellar functions	
Normal	0
Mild cerebellar ataxia (associated with truncal sway or hypermetria)	1
Mild or moderate cerebellar ataxia alongside tremors (associated with truncal sway or hypermetria)	2
Severe cerebellar ataxia (associated with tremors, truncal sway, or hypermetria)	3
Brainstem functions	
Normal	0
Mild disability on cranial nerve assessment (facial nerve dysfunction and/or positional pathological nystagmus)	1
Moderate disability on cranial nerve assessment (trigeminal nerve dysfunction, hypoglossal nerve dysfunction, and/or persistent pathological nystagmus)	2
Severe disability on cranial nerve assessment (dysphagia and/or laryngeal dysfunction)	3
Visual functions	
Normal	0
Reduced menace response in 1 eye with impaired vision	1
Absent menace response in 1 eye with impaired vision (other eye normal or reduced but present menace response) or reduced but present menace response in both eyes with impaired vision	2
Absent menace response in both eyes with impaired vision	3
Postural abnormalities	
Normal	0
Head tilt or head turn	1
Pleurothotonus (head and body turn) or head tilt and head turn	2
Decerebellate or decerebrate rigidity	3
Seizure (within the previous 7 days)	
None	0
Controlled seizures	1
Cluster seizures or refractory seizures^a^	2
Status epilepticus	3

*Note*: The scale relies on attributing a numerical rating of dysfunction (0‐3) in 7 categories giving an overall score of between 0 (normal) and a theoretical maximum of 21 (severe disability).

^a^ Refractory seizures were defined as requiring dose increase of any antiepileptic drug or additional treatment with a second medication.

The ICC values for prospective and retrospective use of the NDS (after removal of the binary categories) are presented in Table 3. The prospective results showed good interobserver agreement. Retrospective use showed good agreement between both retrospective reviewers and moderate agreement between prospective and retrospective data collection.

**TABLE 3:** Intraclass correlation coefficients (with 95% confidence intervals in parenthesis) for interobserver agreement of the neurodisability scale (NDS) total score recorded by 2 independent assessors and for the retrospective assessor.

	Prospective interobserver agreement	Retrospective interobserver agreement	Agreement between retrospective and prospective observers
NDS total score	0.830 (0.679‐0.914)	0.843 (0.701‐0.921)	0.706 (0.562‐0.826)

Median follow‐up time was 11 months (IQR 8‐21). Four dogs did not survive to discharge: 3 dogs were euthanized in the hospital because of difficulty controlling status epilepticus (SE) and 1 dog suffered cardiorespiratory arrest during hospitalization. Four cases were excluded from long‐term outcome analysis; 2 were euthanized (2‐12 weeks after diagnosis) without adequate treatment because of financial constraints, 1 was euthanized 2 weeks after diagnosis because of development of pancreatitis, and 1 was lost to follow‐up 1 month after diagnosis. Long‐term outcome was good in 8 cases, fair in 8 cases, and poor in 11 cases. Fourteen dogs experienced clinical relapse, with a median time to relapse of 7 months (IQR, 3‐12). Of these, 7 responded appropriately to changes in treatment (median time to relapse, 12 months; IQR, 9‐17) whereas 7 showed no or minimal improvement (median time to relapse, 3 months; IQR, 2‐5). A significant difference in time to relapse was found between dogs that responded and those that did not respond to treatment (*P* = .01). No association was found between NDS score and outcome (*P* = .24).

A significant difference (*P* = .02) was found between the NDS score of dogs that survived to discharge (median, 8; range, 3‐14) compared to those that did not (median, 12.3; range, 8‐13). A significant difference (*P* = .01) also was found between the ICU hospitalization time of dogs that survived to discharge (median, 1 day; range, 0‐7) compared to those that did not (median, 5 days; range, 2‐7). The NDS score also was associated with ICU hospitalization time (*r* = 0.41, *P* = .02).

## 4 DISCUSSION

The NDS was designed, consistent with clinical outcome scales used in inflammatory CNS diseases in humans and based on our retrospective study of neurological examination findings, as an objective clinician‐administered measure of neurological impairment in dogs with MUO. The scale relies on attributing a numerical rating of dysfunction (0‐3) in 7 categories, giving an overall score of between 0 (normal) and a theoretical maximum of 21 (severe disability). We found good agreement between assessors when using the NDS prospectively, but only moderate agreement was found between prospective and retrospective use. No association was found between the NDS score at admission and long‐term outcome, but the score was associated with survival to discharge and days spent in the ICU. No association was found between the NDS score and relapse, but patients that relapsed earlier were less likely to respond to treatment.

Development and validation of clinical outcome measures (COMs) in veterinary medicine enable standardized assessment of a patient's disease status and provide a better measure of the efficacy of treatment in clinical trials. A useful COM should have sound psychometric properties including reliability, validity, and responsiveness.^16^, ^23^, ^24^ Reliability is the degree of consistency exhibited when a measurement is repeated under identical conditions.^23^, ^24^ Our results indicated high interobserver reliability for the prospective use of the total NDS score and most of the categories that compose it, except for the 2 binary categories (proprioceptive deficits and hyperesthesia), which therefore were withdrawn from the final NDS. Some of the discrepancies identified might be a result of variation in experience among assessors and also time spent with the patients; the latter of which was often different because typically 1 of the assessors was the clinician responsible for the patient whereas the other may have only performed the neurological examination and completed the NDS score sheet.

Unfortunately, validity and responsiveness could not be assessed in our study. Validity refers to how well a test measures what it was developed to evaluate.^23^, ^24^, ^25^ The validity of an instrument can be examined in several ways, but the 3 most common subtypes are content, criterion, and construct validity. Content validity is the extent to which an instrument measures the variety of attributes that make up the desired construct.^23^, ^24^, ^25^ It most commonly relies on groups of experts in the particular discipline who review the COM and approve it or recommend changes. Criterion validity refers to the extent to which the measure agrees with the external standard measure, but it cannot be assessed in MUO because no such measure currently exists. Construct validity represents the extent to which the instrument accurately assesses what it is designed to evaluate. It is evaluated by how the construct correlates with similar measures but, unfortunately, no other COMs are currently available for MUO. Responsiveness measures an instrument's ability to capture change.^23^, ^24^, ^25^ Future studies with larger numbers of dogs are required to further validate the NDS and, most importantly, to evaluate its responsiveness. Such studies will provide further support as to whether the NDS can be a useful tool in monitoring disease progression in patients with MUO and to assess the effectiveness of therapeutic interventions in clinical trials.

When using clinical assessment scales, methods should be used to increase reliability, including training of investigators, assessment by the same rater during the study, standardized protocols for neurological examination, and precise definitions of all requirements.^17^ Most raters in our study used the scale on several occasions but some (mainly the rotating interns) were less experienced in performing neurological examinations and only used the NDS on a single occasion. Nonetheless, inter‐rater reliability was high, suggesting that the NDS is a robust COM. Increasing reliability further by using the same assessor throughout a trial should be considered in future studies. Using the same COM (such as the NDS) in different studies of MUO may overcome the limitations of survival as an outcome measure and could allow for easier comparison of results.

In our initial study, outcome (defined as good, fair, or poor) was not associated with NDS score. The NDS may not discriminate sufficiently among different severities of clinical signs, but it possibly also is affected by the different pathologies included within a clinical diagnosis of MUO and the absence of a standardized treatment protocol. It is also likely that it could be related to small sample size. Post hoc power analysis based on the effect size identified by our study using 3 outcome groups (with a significance level of .05), identified statistical power of 0.18. Previous studies have identified that younger age at diagnosis^9^ and early diagnosis (within 7 days of development of clinical signs)^14^ were associated with longer survival times whereas seizures or altered mentation were associated with shorter survival times.^5^, ^22^, ^26^

Although we should be cautious interpreting the findings relating to relapse because only 14 dogs were in this group, a significant difference was identified in terms of response to treatment, with those that relapsed earlier in the treatment course less likely to respond to rescue protocols. In a disease that is often fatal and associated with poor long‐term outcome,^1^, ^5^, ^7^ the need for biomarkers that could be used to monitor disease progression and response to treatment is very important. Recently, serum neurofilament light chain (NfL) concentration has been shown to decrease over time in dogs with MUO that show good response to treatment and to increase significantly in those that experience poor response to treatment.^13^ This result is promising, but unfortunately this test is not yet widely available commercially. The use of COMs to monitor dogs with MUO may help clinicians identify signs of poor response to treatment earlier and allow adjustment of treatment protocols accordingly. Future studies assessing the responsiveness of the NDS are underway to investigate if the NDS could be a useful tool for monitoring disease progression over time and whether it will have a positive effect on the management of these patients, potentially improving long‐term outcomes.

Most clinical studies in veterinary medicine rely on retrospective data. It therefore would be very beneficial to be able to use COMs such as the NDS through retrospective review of medical records rather than prospectively collecting this information. Our results showed moderate agreement between retrospective and prospective use of the NDS, suggesting that this approach may be possible, but such use should be undertaken with caution and accepting lower reliability as compared to prospective data collection. The retrospective data collection in our study was based mostly on referral letters written by trained neurologists summarizing their findings. These could have been more detailed than usual because the clinician had completed the NDS at admission, and less information may have been recorded if the NDS had not been used. The reliability of the retrospective use of the NDS likely will vary depending on the quality of the clinical records.

Obtaining the NDS score in patients in SE is understandably challenging because, in most cases, these dogs are unconscious, recumbent, and receiving multiple sedative medications. In such cases, a maximum score of 3 was assigned in the ambulatory, cerebral, vision, and seizure categories and 0 in all other categories by all assessors independently. Deficits in other categories might have been present in those patients, because MUO commonly causes multifocal disease and it is likely that animals would not have had the maximum severity for some of those categories where they were attributed. Nonetheless, seizures were present in >33% of the initial population and these patients often presented with cluster seizures or SE, and thus we felt it was important to include them in the study. The presence of seizures as part of the clinical presentation has been suggested to be associated with worse outcome in previous studies.^5^, ^22^ It was therefore considered valuable to include the seizure category, not only as it may affect outcome but also because epilepsy can be debilitating and substantially affect the quality of life of both the dog and its owner.^27^, ^28^, ^29^

Our study had some limitations. Most importantly, we could not assess the validity or responsiveness of the NDS. No other COMs related to MUO are available and thus assessing validity is difficult, but consulting a wider group of experienced veterinary neurologists during scale design could have been useful. We also could not evaluate intraobserver reliability because immediately after initial assessment patients underwent general anesthesia for diagnostic procedures and shortly after received immunosuppressive treatment. Consequently, different time points could not be reliably used. The treatment protocol varied (after the initial treatment which was the same for all patients) and often was guided by owner preference (often based on time restrictions to attend hospital consultations and financial considerations). It is likely that these factors affected long‐term outcome although there is currently no gold standard treatment for MUO because of a lack of double‐blinded prospective studies comparing different treatment options. The population used to inform scale design and that used for subsequent assessment of scale reliability should be similar, but in our study included a different breed distribution. Unfortunately, this design feature was not something that we could control and reflects the different popularity of dog breeds over time. The inherent subjectivity of the neurological examination itself and the use of terminology such as mild and moderate to describe degrees of severity in some of the categories of the NDS likely also contributed to higher variability. These are limitations characteristic of COMs that rely on subjective criteria and are susceptible to subjective interpretation by clinicians. Some subjective decisions on which neurological deficits should be attributed to the different degrees of dysfunction were made during development of the scale, mostly relating to signs of cerebral and brainstem dysfunction. These were undertaken based on the most common neurological signs identified on the initial study population and what effects they would have on quality of life and possible resulting disability. Although some subjectivity was introduced during scale design, providing specific examples to each degree of dysfunction should decrease variability for users of the scale thereby improving its reliability. The time at last follow‐up varied among patients because the last outcome information was collected immediately before data analysis. This time was not standardized and likely influenced the results despite a minimum follow‐up time of 6 months and cases with inappropriate treatment having been excluded from outcome analysis. Dogs that did not undergo CSF analysis because of suspicion of increased ICP on MRI were not excluded from analysis because doing so likely would have biased the data by excluding the most severely affected dogs. Nonetheless, this design feature also may have added bias if those dogs were misdiagnosed because this information was missing. Some dogs had received different medications before examination (including corticosteroids in 4 cases), which likely affected the NDS score at presentation but is representative of clinical practice in which the NDS would be used routinely. Lastly, this scale cannot be used in dogs with meningoencephalomyelitis of unknown origin and neurological deficits associated with spinal cord disease only. Meningoencephalomyelitis can in some cases result only in clinical signs attributable to spinal cord disease (most commonly ambulatory paraparesis)^30^ and in such circumstances, a spinal cord injury scale could be used for monitoring.^31^, ^32^, ^33^

## 5 CONCLUSION

The NDS is a novel clinical scale for recording the degree of disability in MUO and has shown good reliability. It could be used in clinical practice and may help overcome current limitations in monitoring MUO patients and determining outcomes in therapeutic trials.

## CONFLICT OF INTEREST DECLARATION

Authors declare no conflict of interest.

## OFF‐LABEL ANTIMICROBIAL DECLARATION

Authors declare no off‐label use of antimicrobials.

## INSTITUTIONAL ANIMAL CARE AND USE COMMITTEE (IACUC) OR OTHER APPROVAL DECLARATION

Authors declare no IACUC or other approval was needed.

## HUMAN ETHICS APPROVAL DECLARATION

Authors declare human ethics approval was not needed for this study.

## Supporting information


**Data S1.** Supporting Information.

## References

[1] Cornelis I , Van Ham L , Gielen I , et al. Clinical presentation, diagnostic findings, prognostic factors, treatment and outcome in dogs with meningoencephalomyelitis of unknown origin: a review. Vet J. 2019;244:37‐44. 30825893 10.1016/j.tvjl.2018.12.007

[2] Coates JR , Jeffery ND . Perspectives on meningoencephalomyelitis of unknown origin. Vet Clin North Am Small Anim Pract. 2014;44:1157‐1185. 25239815 10.1016/j.cvsm.2014.07.009

[3] Talarico LR , Schatzberg SJ . Idiopathic granulomatous and necrotising inflammatory disorders of the canine central nervous system: a review and future perspectives. J Small Anim Pract. 2010;51:138‐149. 19814766 10.1111/j.1748-5827.2009.00823.x

[4] Gonçalves R , De Decker S , Walmsley G , et al. Inflammatory disease affecting the central nervous system in dogs: a retrospective study in England (2010‐2019). Front Vet Sci. 2021;8:819945. 35155652 10.3389/fvets.2021.819945 PMC8829331

[5] Cornelis I , Volk HA , Van Ham L , et al. Prognostic factors for 1‐week survival in dogs diagnosed with meningoencephalitis of unknown aetiology. Vet J. 2016;214:91‐95. 27387733 10.1016/j.tvjl.2016.05.008

[6] Lowrie M , Smith PM , Garosi L . Meningoencephalitis of unknown origin: investigation of prognostic factors and outcome using a standard treatment protocol. Vet Rec. 2013;172:527. 23462382 10.1136/vr.101431

[7] Muñana KR , Luttgen PJ . Prognostic factors for dogs with granulomatous meningoencephalomyelitis: 42 cases (1982‐1996). J Am Vet Med Assoc. 1998;212:1902‐1906. 9638190

[8] Lowrie M , Thomson S , Smith P , Garosi L . Effect of a constant rate infusion of cytosine arabinoside on mortality in dogs with meningoencephalitis of unknown origin. Vet J. 2016;213:1‐5. 27240905 10.1016/j.tvjl.2016.03.026

[9] Oliphant BJ , Barnes Heller HL , White JM . Retrospective study evaluating associations between midline brain shift on magnetic resonance imaging and survival in dogs diagnosed with meningoencephalitis of unknown etiology. Vet Radiol Ultrasound. 2017;58:38‐43. 27774741 10.1111/vru.12434

[10] Cornelis I , Volk HA , De Decker S . Clinical presentation, diagnostic findings and long‐term survival in large breed dogs with meningoencephalitis of unknown aetiology. Vet Rec. 2016;179:147. 27165997 10.1136/vr.103640

[11] Levine JM , Fosgate GT , Porter B , Schatzberg SJ , Greer K . Epidemiology of necrotizing meningoencephalitis in Pug dogs. J Vet Intern Med. 2008;22:961‐968. 18647157 10.1111/j.1939-1676.2008.0137.x PMC7166975

[12] Portero M , Martínez de Merlo E , Pérez C , Benito M , Daza MA , Fragio C . Cerebrospinal fluid and blood lactate concentrations as prognostic biomarkers in dogs with meningoencephalitis of unknown origin. Vet J. 2019;254:105395. 31836169 10.1016/j.tvjl.2019.105395

[13] Yun T , Koo Y , Chae Y , et al. Neurofilament light chain as a biomarker of meningoencephalitis of unknown etiology in dogs. J Vet Intern Med. 2021;35:1865‐1872. 34114244 10.1111/jvim.16184 PMC8295659

[14] Barnoon I , Shamir MH , Aroch I , et al. Retrospective evaluation of combined mycophenolate mofetil and prednisone treatment for meningoencephalomyelitis of unknown etiology in dogs: 25 cases (2005‐2011). J Vet Emerg Crit Care (San Antonio). 2016;26:116‐124. 26458162 10.1111/vec.12399

[15] Smith PM , Stalin CE , Shaw D , Granger N , Jeffery ND . Comparison of two regimens for the treatment of meningoencephalomyelitis of unknown etiology. J Vet Intern Med. 2009;23:520‐526. 19645837 10.1111/j.1939-1676.2009.0299.x

[16] Inojosa H , Schriefer D , Ziemssen T . Clinical outcome measures in multiple sclerosis: a review. Autoimmun Rev. 2020;19:102512. 32173519 10.1016/j.autrev.2020.102512

[17] Meyer‐Moock S , Feng YS , Maeurer M , Dippel FW , Kohlmann T . Systematic literature review and validity evaluation of the Expanded Disability Status Scale (EDSS) and the multiple sclerosis functional composite (MSFC) in patients with multiple sclerosis. BMC Neurol. 2014;14:58. 24666846 10.1186/1471-2377-14-58 PMC3986942

[18] Greer KA , Wong AK , Liu H , et al. Necrotizing meningoencephalitis of Pug dogs associates with dog leukocyte antigen class II and resembles acute variant forms of multiple sclerosis. Tissue Antigens. 2010;76:110‐118. 20403140 10.1111/j.1399-0039.2010.01484.x

[19] Stafford EG , Kortum A , Castel A , et al. Presence of cerebrospinal fluid antibodies associated with autoimmune encephalitis of humans in dogs with neurologic disease. J Vet Intern Med. 2019;33:2175‐2182. 31495976 10.1111/jvim.15616 PMC6766506

[20] Kurtzke JF . On the origin of EDSS. Mult Scler Relat Disord. 2015;4:95‐103. 25787185 10.1016/j.msard.2015.02.003

[21] Lim JA , Lee ST , Moon J , et al. Development of the clinical assessment scale in autoimmune encephalitis. Ann Neurol. 2019;85:352‐358. 30675918 10.1002/ana.25421

[22] Granger N , Smith PM , Jeffery ND . Clinical findings and treatment of non‐infectious meningoencephalomyelitis in dogs: a systematic review of 457 published cases from 1962 to 2008. Vet J. 2010;184:290‐297. 19410487 10.1016/j.tvjl.2009.03.031

[23] Boateng GO , Neilands TB , Frongillo EA , Melgar‐Quiñonez HR , Young SL . Best practices for developing and validating scales for health, social, and behavioral research: a primer. Front Public Health. 2018;6:149. 29942800 10.3389/fpubh.2018.00149 PMC6004510

[24] Narayanaswami P . The spectrum of functional rating scales in neurology clinical trials. Neurotherapeutics. 2017;14:161‐175. 27796917 10.1007/s13311-016-0488-5 PMC5233630

[25] Kimberlin CL , Winterstein AG . Validity and reliability of measurement instruments used in research. Am J Health Syst Pharm. 2008;65:2276‐2284. 19020196 10.2146/ajhp070364

[26] Bateman SW , Parent JM . Clinical findings, treatment, and outcome of dogs with status epilepticus or cluster seizures: 156 cases (1990‐1995). J Am Vet Med Assoc. 1999;215:1463‐1468. 10579043

[27] Wessmann A , Volk HA , Packer RM , et al. Quality‐of‐life aspects in idiopathic epilepsy in dogs. Vet Rec. 2016;179:229. 27329504 10.1136/vr.103355

[28] Packer RM , Volk HA . Epilepsy beyond seizures: a review of the impact of epilepsy and its comorbidities on health‐related quality of life in dogs. Vet Rec. 2015;177:306‐315. 26405128 10.1136/vr.103360

[29] Nettifee JA , Munana KR , Griffith EH . Evaluation of the impacts of epilepsy in dogs on their caregivers. J Am Anim Hosp Assoc. 2017;53:143‐149. 28291397 10.5326/JAAHA-MS-6537

[30] Cornelis I , Volk HA , Van Ham L , et al. Clinical presentation, diagnostic findings and outcome in dogs diagnosed with presumptive spinal‐only meningoen‐cephalomyelitis of unknown origin. J Small Anim Pract. 2017;58:174‐182. 28267222 10.1111/jsap.12622 PMC7166691

[31] Olby NJ , De Risio L , Muñana KR , et al. Development of a functional scoring system in dogs with acute spinal cord injuries. Am J Vet Res. 2001;62:1624‐1628. 11592330 10.2460/ajvr.2001.62.1624

[32] Levine GJ , Levine JM , Budke CM , et al. Description and repeatability of a newly developed spinal cord injury scale for dogs. Prev Vet Med. 2009;89:121‐127. 19303151 10.1016/j.prevetmed.2009.02.016

[33] Lee CS , Bentley RT , Weng HY , Breur GJ . A preliminary evaluation of the reliability of a modified functional scoring system for assessing neurologic function in ambulatory thoracolumbar myelopathy dogs. BMC Vet Res. 2015;11:241. 26403185 10.1186/s12917-015-0557-8 PMC4583166

