# Supplementary material for: Development of a reliable clinical assessment tool for meningoencephalitis in dogs: The neurodisability scale
Source: J Vet Intern Med. 2023 Apr 24;37(3):1111–8. doi: 10.1111/jvim.16717 (PMC10229334; doi:10.1111/jvim.16717)
Supplement: Supplementary file 1 — Data S1. Supporting Information. [file JVIM-37-1111-s001.pdf]

Supplementary Table 1. Initial generated canine meningoencephalitis neurodisability scale

	Score
Ambulatory status	
normal	0
mild paresis or ataxia present but ambulatory without falling	1
moderate/severe paresis or ataxia present with frequent falling	2
non-ambulatory	3
Cerebral functions	
normal	0
disorientation, obtundation or behaviour changes (only 1 of these abnormalities)	1
2 or all of following: disorientation, obtundation or behaviour changes	2
stupor, coma, compulsive circling and/or head pressing (+/- disorientation, behavior changes or obtundation)	3
Cerebellar functions	
normal	0
mild cerebellar ataxia (associated with truncal sway or hypermetria)	1
mild or moderate cerebellar ataxia and tremors (associated with truncal sway or hypermetria)	2
severe cerebellar ataxia (associated with tremors, truncal sway or hypermetria)	3
Brainstem functions	
normal	0
mild disability on cranial nerve assessment (facial nerve dysfunction and/or positional pathological nystagmus)	1
moderate disability on cranial nerve assessment (trigeminal nerve dysfunction, hypoglossal nerve dysfunction and/or persistent pathological nystagmus)	2
severe disability on cranial nerve assessment (dysphagia and/or laryngeal dysfunction)	3
Visual functions	
normal	0
reduced menace response in one eye with impaired vision	1
absent menace response in one eye with impaired vision (other eye normal or reduced but present menace response) or reduced but present menace response in both eyes with impaired vision	2
absent menace response in both eyes with impaired vision	3
Postural abnormalities	
normal	0
head tilt or head turn	1
pleurothotonus (head and body turn) or head tilt and head turn	2
decerebellate or decerebrate rigidity	3
Seizure	
none	0
controlled seizures	1
refractory seizures*	2
Status Epilepticus or cluster seizures	3
Hyperesthesia (head or spinal)	
absent	0
present	1
Proprioceptive deficits	
absent	0
present in any limb	1

*Refractory seizures were defined as requiring dose increase of any antiepileptic drug or additional treatment with a second medication

Supplementary Table 2. Linear weighted kappa (with 95% confidence intervals in parenthesis) for inter-observer agreement of ordinal categories and Cohen's kappa for the binary categories recorded by two independent assessors.

	**Prospective inter-observer agreement**
**Seizures**	1<br>(1-1)
**Ambulatory status**	0.776<br>(0.609-0.944)
**Cerebral functions**	0.608<br>(0.421-0.794)
**Cerebellar functions**	0.752<br>(0.529-0.975)
**Brainstem functions**	0.583<br>(0.36-0.806)
**Visual functions**	0.733<br>(0.525-0.94)
**Postural abnormalities**	0.689<br>(0.484-0.895)
**Hyperaesthesia**	0.294<br>(0-0.633)
**Proprioception deficits**	0.125<br>(0-0.507)
